# Regulation of vascular signalling by nuclear Sprouty2 in fetal lung epithelial cells: Implications for co-ordinated airway and vascular branching in lung development

**DOI:** 10.1016/j.cbpb.2018.01.007

**Published:** 2018-10

**Authors:** David J. Walker, Stephen C. Land

**Affiliations:** D'Arcy Thomson Unit, Biological and Biomedical Science Education, School of Life Sciences, University of Dundee, Dundee, DD1 4HN, Scotland, UK.

**Keywords:** Lung development, Gas exchange, Cardio-pulmonary system, Hypoxia inducible factor, Fibroblast growth factor-10, Symmorphosis

## Abstract

Sprouty2 (Spry2) acts as a central regulator of tubular growth and branch patterning in the developing mammalian lung by controlling both magnitude and duration of growth factor signalling. To determine if this protein coordinates airway and vascular growth factor signalling, we tested the hypothesis that Spry2 links the primary cue for airway outgrowth, fibroblast growth factor-10 (FGF-10), to genomic events underpinning the expression and release of vascular endothelial growth factor-A (VEGF-A). Using primary fetal distal lung epithelial cells (FDLE) from rat, and immortalised human bronchial epithelial cells (16HBE14o-), we identified a nuclear sub-population of Spry2 which interacted with regions of the rat and human VEGF-A promoter spanning the hypoxia response element (HRE) and adjacent 3′ sites. In FDLE cultured at the PO_2_ of the fetal lung, FGF-10 relieved the Spry2 interaction at the HRE region by promoting clearance of a 39 kDa form and this was accompanied by histone-3 S10K14 phosphoacetylation, promoter de-methylation, hypoxia inducible factor-1α activation and VEGF-A expression. This repressive characteristic of nuclear Spry2 was relieved in 16HBE14o- by shRNA knockdown, and stable expression of mutants (C218A; C221A) that do not interact with the VEGF-A promoter HRE region. We conclude that nuclear Spry2 acts as a molecular link which co-ordinates airway and vascular growth of the cardiopulmonary system. This identifies Spry2 as a contributing determinant of design optimality in the mammalian lung.

## 1 Introduction

Weibel's latex cast of the human pulmonary airway and vascular tree vividly illustrates the problem of optimality in anatomical design and physiological function (the Symmorphosis debate; see image in Glenny (2011)). We see an interwoven network of airway, arterial and venous tubes branching at regular intervals with diminishing orders of magnitude for up to ~17 generations, after which the morphogenic programme diverges to create the alveoli and capillary bed of the respiratory acinus. For all that this structure creates a low resistance/high surface area for gas exchange, aerobic performance is constrained by one fundamental design limitation: a single path for convective gas movement to and from the blood-gas barrier. To be viable, this system requires extraordinary scalar proportions (Glenny, 2011; Weibel, 1991) which are generated by the interlinked branching morphogenesis programme of the airway and vascular systems and it is this process which ultimately sets the physiological boundaries for cardiovascular performance. Peter Hochachka was a career-long contributor to the Symmorphosis debate (e.g. (Hochachka et al., 1998)) and many of the biochemical structure/function relationships he explored arise as adaptive responses to the cardio-pulmonary system under challenge. In recognition of his contributions to the field, this original research article presents molecular evidence to support a role for Sprouty2 as a co-ordinating link between the airway and vascular branching morphogenesis programmes. As such, we propose Sprouty2 as a key contributing factor in establishing structure-function optimality in gas exchange systems.

Sprouty (Spry) proteins play a key role in organ development by defining the temporal and spatial patterning of growth events in folded and branched organ structures. Early studies in *Drosophila* demonstrated that knockout of the single *Spry* gene enhanced branching rate of the tracheal airway (Hacohen et al., 1998) and later work in mammalian lung development established roles for a family of Spry homologs (*Spry1-4*) in the regulation of airway tube elongation and branching (de Maximy et al., 1999; Scott et al., 2010; Tefft et al., 2005; Tefft et al., 2002; Tefft et al., 1999; Perl et al., 2003). The evidence suggests that Sprouty proteins participate in the morphogenic subroutines which determine fractal patterning in branched systems.

In lung development, airway branching is induced by mesoderm-derived fibroblast growth factor-10 (FGF-10) binding to the tyrosine kinase receptor, FGFR2b, expressed in cells at the tip of the endodermal airway tube. Downstream signalling through extracellular regulated kinase 1/2 (ERK1/2) and the mechanistic target of rapamycin complex 1 (mTORC1) (Scott et al., 2010) induces outward growth toward the FGF-10 signal and the duration of this event, a determinant of branch length in the fractal patterning of the airway tree, is antagonised by Spry2 (Tefft et al., 2002, Tefft et al., 2005). We have expanded this model by showing that Spry2 has the potential to co-ordinate vascular growth with the airway branching programme by activating an mTORC-1–hypoxia inducible factor-1α (HIF-1α) complex which drives vascular endothelial growth factor-A (VEGF-A) expression and secretion from the airway endoderm (Land et al., 2014; Scott et al., 2010). Since publishing this work, however, it has emerged that a sub-population of Spry2 resides in the nucleus (Ahn et al., 2010; Aranda et al., 2008), raising the possibility that this regulator of airway branching could influence nuclear events during the branching process. To determine if Spry2 directly links the primary cues for airway (FGF-10) and vascular (VEGF-A) growth in the pulmonary branching morphogenesis programme, we tested the hypothesis that FGF-10 induces direct interaction with between nuclear Spry2 and key elements of VEGF-A gene regulation in fetal lung endodermal epithelium. Our results show that Spry2 binds at several key positions in the 5′VEGF-A promoter and that FGF-10 modulates this interaction to induce VEGF-A transcript expression. In addition to its well-established role as a regulator of receptor tyrosine kinase activity at the cell surface, we conclude that Spry2 mediates epigenetic events in the nucleus and so co-ordinates gene expression with airway and vascular growth factor signalling intensity.

## 2 Materials and methods

### 2.1 Plasmids and antibodies

N-terminal FLAG and C-terminal Myc dual tagged Spry2 was constructed by sub-cloning full length Spry2 into pCMV-Tag-1 (Stratagene, La Jolla CA, USA) and cysteine to alanine mutations were generated at positions 218 and 221 to assess Spry2-DNA interactions. Non-genome target control (TR20003) and shRNA vectors targeting human Spry2 in pRS were originally purchased from Origene Technologies, Inc. (Rockville, MD; Catalog number TR309130) and kindly supplied to us by Drs P Yusoff and G Guy (Proteos, Singapore). Stable Spry2 knockdown in 16HBE14o- cells was achieved with the clone TI336513 (sequence ‘5-CTGAACAGAGACTGCTAGGATCATCCTTC-3’). Source, concentration and species of all antibodies used in this study are given in Supplementary Table 1.

### 2.2 Cell culture and manipulation

Primary fetal distal lung epithelial cells (FDLE) were isolated from gestation day 19 Sprague Dawley rat lungs at the pseudoglandular/canalicular stage of development and maintained in polarised culture on permeable supports as described (Land and Collett, 2001; Scott et al., 2010). Culture of the human bronchial epithelial cell line (16HBE14o- or “HBE” (Cozens et al., 1994)) was also as described (Scott et al., 2010). Unless stated, cells were maintained at PO_2_ equivalent to the fetal lung in utero (3% O_2_) to conserve the FGF-10 and HIF signalling pathway context (Scott et al., 2010). HBE stably transformed with dual-tagged Spry2, Spry2-C218A or Spry2-C221A were generated by puromycin selection of clonal cells.

### 2.3 Preparation of cytosolic, nuclear and chromatin-bound lysates

The full protocol is given in Supplementary Fig. 2 with initial lysis procedures were conducted in a hypoxic chamber equilibrated to 3% O_2_. Enrichment of Histone 3 (H3) confirmed nuclear lysis.

### 2.4 Electrophoretic mobility shift assay (EMSA)

Protein interactions with the HIF DNA consensus sequence were performed using [^32^P] radiolabelled oligonucleotides containing the HIF response element (HRE) in the rat VEGF promoter (HRE underlined): 5′-TGC ATA CGT GGG CTT CCA CAG GTC-3′. The same sequence with the HRE mutated to adenosines served as a negative control. Nuclear protein binding and mobility shift were performed as before (Land and Darakhshan, 2004) on 8% gels (37.5:1 acrylamide:bis-acrylamide). Supershift assays were performed by pre-incubating nuclear protein with 1 μg of antibody on ice for 1 h before addition of probe. Supershifts were resolved in 6% gels (29:1 acrylamide:bis-acrylamide).

### 2.5 Western blotting, VEGF ELISA and immunofluorescence

Performed as described (Scott et al., 2010) using antibodies given in the respective figure legend.

### 2.6 Quantitative real-time PCR

RNA was isolated using a Nucleospin RNA II Kit (Macherey-Nagel). 1 μg of RNA was reverse-transcribed using a Quantitect Reverse Transcriptase Kit (Qiagen). qPCR was performed in duplicate on Rotor-Gene Q cycler using a Rotor-Gene SYBR Green PCR Kit using Quantitect Primer Assays (Qiagen). β_2_ microglobulin or 18S were used as housekeeping genes for reference using the ΔΔCt method. Reaction efficiency, standard curve regression and product melt characteristics were monitored to ensure reaction fidelity.

### 2.7 Chromatin immunoprecipitation assay (ChIP)

The full protocol including primer sets and chIP validation of Anti-Spry2 (Abcam Ab50317) is given in Supplementary Fig. 2 and Table 2.

### 2.8 DNA methylation and high resolution melt analysis

Genomic DNA from wild-type or stably transformed HBE cells was bisulfite converted using the Epitect Fast DNA Bisulfite Kit (Qiagen) according to the manufacturer's instructions. Bisulfite-converted DNA was adjusted to 50 ng/μl after a clean-up step and this volume was used in conjunction with Epitect High Resolution Melting PCR (Qiagen) to detect differences in DNA methylation at CpG sites located 145 bp 5′ to the VEGF-A transcription initiation site (TIS) or 179 bp 3′ to this locus. Methyl Primer Express® Software v1.0 (Applied Biosystems) was used to select CpG methylation-specific primer sets. MS-HRM was performed on a Rotorgene 6000 (Qiagen) and once each sample had reached the plateau phase of the PCR a high resolution melting protocol was used to expose methylation specific differences in the amplified DNA.

### 2.9 Statistics

Multiple comparisons were conducted using one way ANOVA with post hoc significance determined using Tukey's HSD. Values are mean ± SEM with the number of observations reported in the legend to each figure.

## 3 Results

### 3.1 Spry2 is a nuclear protein in primary fetal airway epithelial cells

The first aim of this study was to characterise nuclear Spry2 behaviour in relation to FGF-10 in primary FDLE and immortalised HBE cells. Experiments were conducted at fetal (3% O_2_) as well as ambient (21% O_2_) PO_2_ because fetal development occurs in an oxygen-restricted environment and tissue oxygenation influences total cellular Spry2 stability (Anderson et al., 2011).

**Fig. 1:**
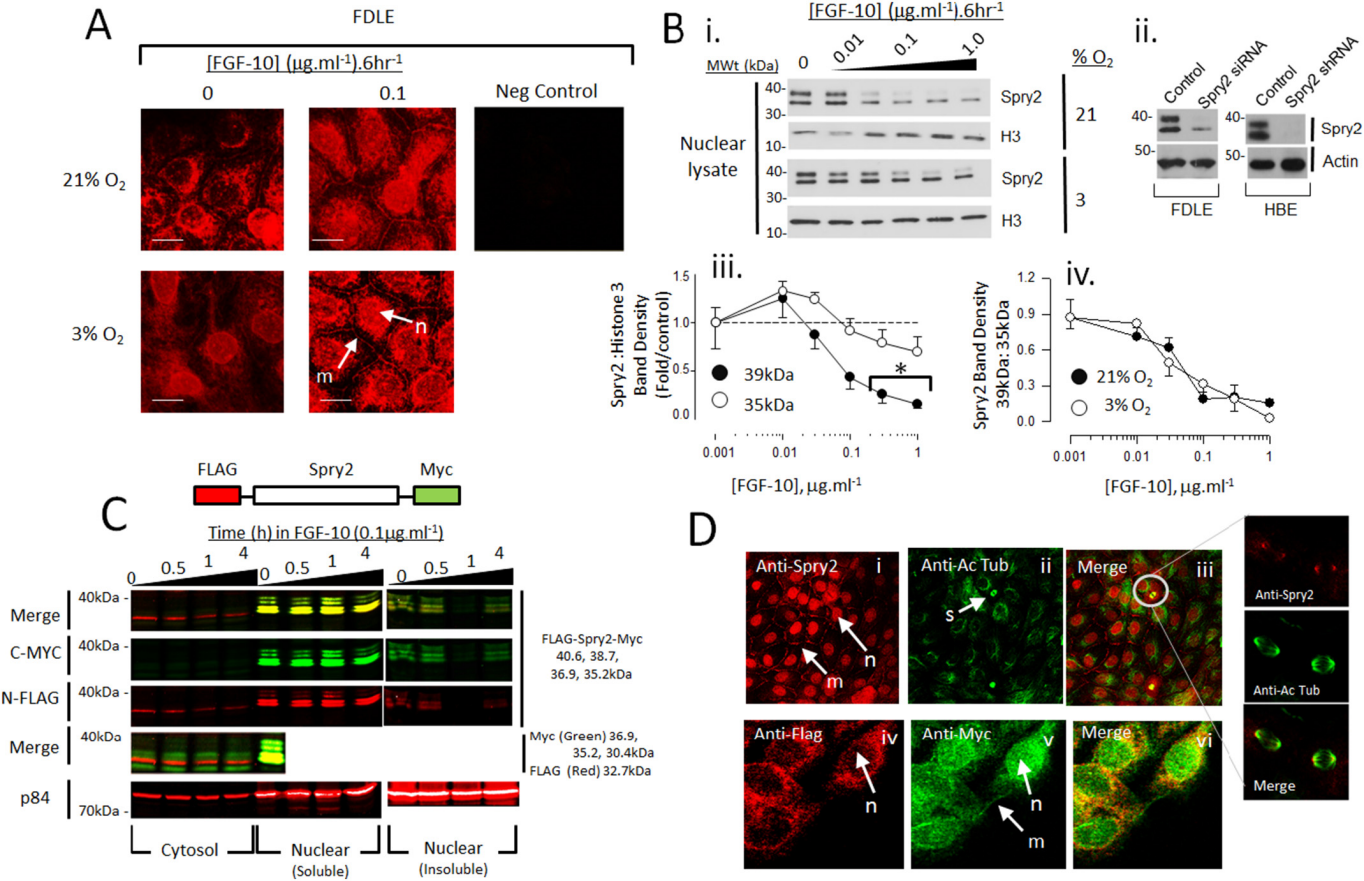
Nuclear expression of Spry2 in FDLE. A. Immunofluorescence shows Spry2 is nuclear in rat FDLE cells and this distribution is conserved irrespective of culture PO_2_ or FGF-10. Image is representative of 4 independent preparations m – membrane, n – nucleus. Neg control, no primary antibody B. Spry2 resolves as 39 and 35 kDa in rat FDLE nuclear lysates; H3, Histone 3 (i). FGF-10 induces clearance 39 kDa Spry2, (ii) Spry2 siRNA (rat FDLE, 250 pmol·ml^−1^) and stable shRNA (HBE) suppresses 35 and 39 kDa Spry2 abundance. (iii) Quantitation of FGF-10 effect upon 39 kDa 35 kDa Spry2 in FDLE, mean ± SEM, n = 6, *P < 0.05 relative to control. (iv) The effect of FGF-10 upon 39 kDa Spry clearance is not altered by culture PO_2_ does not alter irrespective of culture PO_2_, mean ± SEM, n = 6. C. Western blotting of cytosolic and nuclear Spry2 in 16HBE14o- (HBE) cells stably expressing dual tagged Spry2 (upper image). LiCor analysis simultaneously resolved N-terminal FLAG (red) and C-terminal Myc (green) epitope tags in cytosolic, nuclear-soluble and nuclear-insoluble fractions from cells treated for up to 4 h in 0.1 μg·ml^−1^ FGF-10. Yellow in the merged image indicates co-localisation of the tags. The nuclear scaffold protein, p84, served as a loading control. Blots representative of 4 independent experiments. D. Immunofluorescence shows native (i–iii) and dual tagged expressed Spry2 (iv–vi) occurs in the nucleus in HBE cells and associates with the spindle pole in mitotic cells (right). Images representative of 4 independent experiments; Abbreviations: m, membrane; n, nucleus; s, mitotic spindles; Ac Tub, Acetylated α-tubulin. (For interpretation of the references to color in this figure legend, the reader is referred to the web version of this article.)

Immunofluorescence in FDLE revealed that Spry2 nuclear abundance was favoured at 3% O_2_ and resolved into plasma membrane and nuclear populations when exposed to 0.1 μg·ml^−1^ FGF-10 for 6 h (Fig. 1A). Nuclear protein western blots resolved Spry2 as a doublet at 35.4 ± 0.5 and 39.3 ± 0.6 kDa (n = 20) and shRNA knockdown in FDLE and HBE confirmed that both bands were products of the same Spry2 transcript. FGF-10 induced a concentration-dependent clearance of the 39 kDa form over 6 h with an EC_50_ = 84 ± 18 ng·ml^−1^ independently of PO_2_ and no change was observed in the 35 kDa form (Fig. 1Bi–iv). Dual tagged Spry2 expressed in HBE resolved in cytosolic, nuclear soluble and chromatin enriched insoluble fractions Fig. 1C). Immunofluorescence shows native (Fig. 1Di–iii) and dual tagged expressed Spry2 (Fig. 1Div–vi) occurs in the nucleus in HBE and associates with the spindle pole in anaphase cells suggesting a role in planar cell division (Tang et al., 2011).

We investigated the difference in nuclear Spry2 protein mass by determining if either form was influenced by phosphorylation, cleavage or palmitoylation (Ding et al., 2007; Forrester et al., 2011; Scott et al., 2010) (see Supplementary Fig. 1). Interventions in these processes affected both forms equally and so we conclude that both Spry2 forms retain conserved responses to pathways that are known to modify Spry2 activity.

**Fig. 2:**
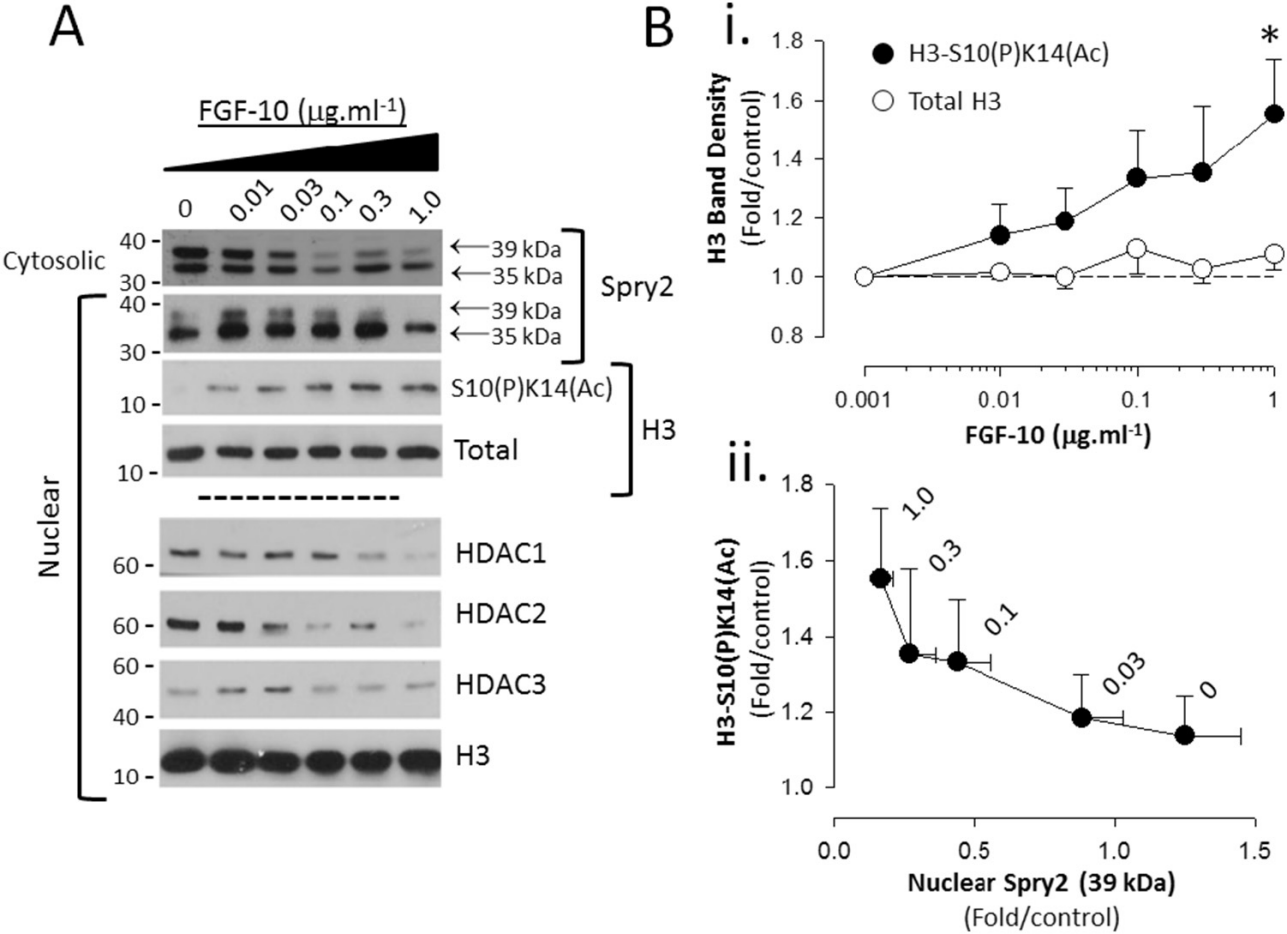
FGF-10 induces histone modification in FDLE and correlates with the decline in 39 kDa Spry2. A. FGF-10-evoked decline in 39 kDa Spry2 in nuclear and cytosolic fractions corresponds with increased Histone 3 S10(P)K14(Ac) phosphoacetylation and decreased nuclear abundance of HDAC1-3. B. (i) Relationship between H3 S10(P)K14(Ac) phosphoacetylation and FGF-10 concentration and (ii) 39 kDa Spry2 abundance (FGF-10 concentration (μg·ml^−1^) shown next to symbols). Values are Mean ± SEM, n = 4; *P < 0.01 relative to control.

To determine if the selected concentration range of FGF-10 induced transcriptional activation we measured histone 3 (H3) phosphoacetylation at serine 10 and lysine 14 (H3-S10(P)K14(Ac)) (Islam and Mendelson, 2008; Karmodiya et al., 2012). FGF-10 induced a concentration dependent increase in H3-S10(P)K14(Ac) phospho-acetylation which was matched by declining HDAC1-3 abundance as well as the 39 kDa Spry2 form (Fig. 2).

### 3.2 Spry2 interacts with the HIF-1α Transcriptional Complex in FDLE and HBE

**Fig. 3:**
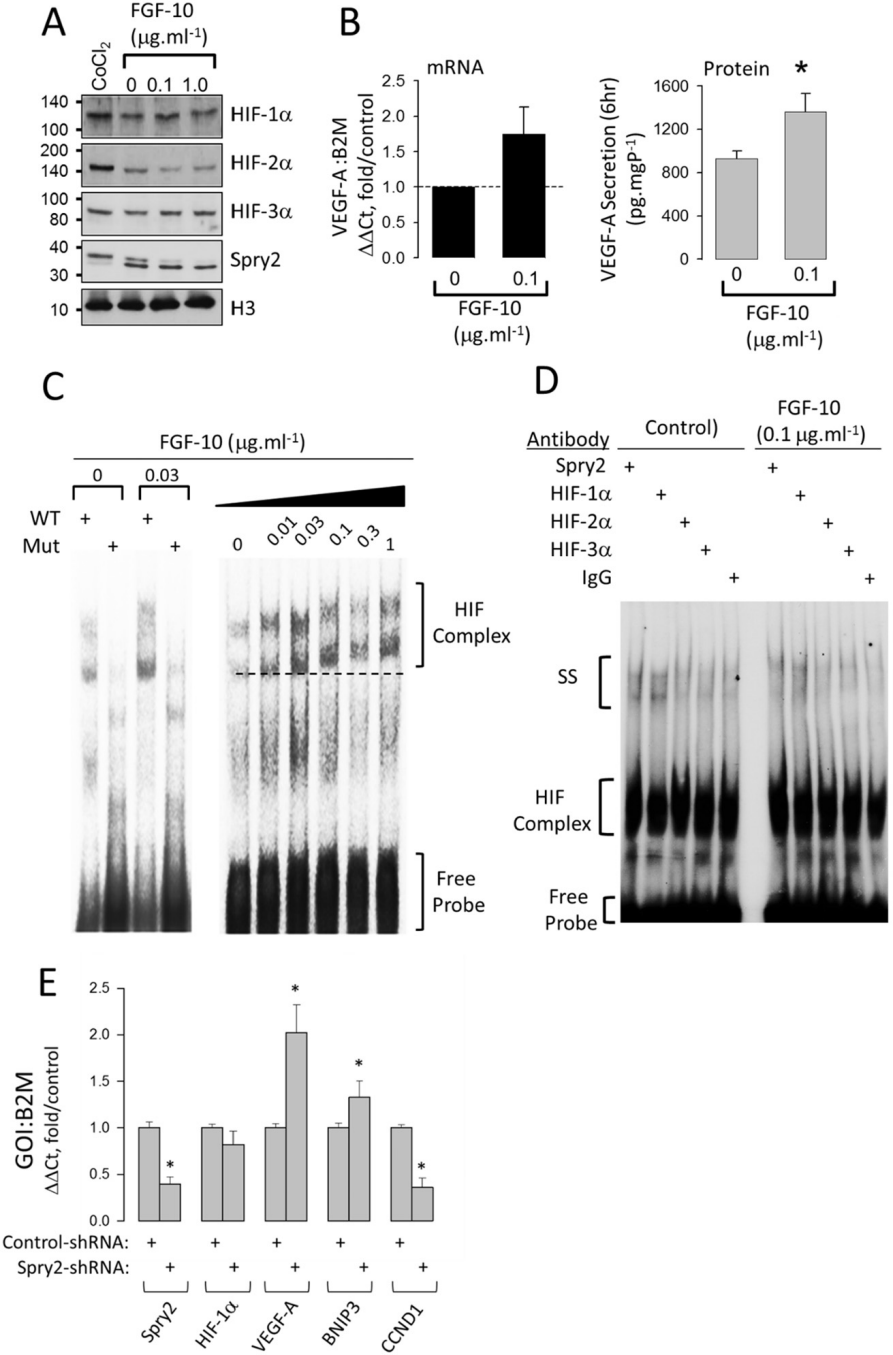
FGF-10 does not alter HIFα subunit stability but increases VEGF-A expression and protein recruitment to the VEGF-A HRE in FDLE. A. Western blot of nuclear HIFα proteins, Spry2 and Histone 3 (H3) from FDLE exposed to increasing concentrations of FGF-10. CoCl_2_ (100 μM, 16 h), positive control, representative of 4 independent experiments. B. mRNA and protein abundance of VEGF-A exposed to FGF-10 measured by qPCR and ELISA, mean ± SEM, n = 4, *P < 0.05. C. EMSA showing retarded migration of [^32^P]-VEGF-A HRE probe incubated with 10 μg of nuclear lysate from FDLE cells treated with FGF-10 as indicated. Mutant probe (Mut) shows non-specific binding; Free probe, unincorporated excess probe; dotted line shows position of band at 0 μg·ml^−1^.FGF-10 D. Supershift EMSA showing decreased mobility of bands incubated with antibodies (1 μg) against Spry2 and HIF-1-3α. Non-immune IgG, control. E. qPCR analysis showing effect of stable Spry2 knockdown in HBE cells (Spry2 shRNA) maintained at fetal PO_2_ upon transcript abundance of Spry2, HIF-1α VEGF-A (regulated by HIF-1α and HIF-2α) BNIP3 (regulated by HIF-1α) and CCND1 (regulated by HIF-2α). Values are mean ± SEM, n = 4, *P < 0.05.

The VEGF-A gene is transcriptionally regulated by HIF-1α and HIF-2α and so we determined which isoform might interact with Spry2 at the VEGF-A promoter. At fetal lung PO_2_, HIF-1, 2 and 3α proteins were detected in the nuclear fraction of FDLE cells and their abundance was not significantly altered by FGF-10 (Fig. 3A). However, VEGF-A mRNA and protein expression were induced by 0.1 μg·ml^−1^ FGF-10 (Fig. 3B) and electrophoretic mobility shift assays (EMSA) revealed enhanced protein recruitment to the VEGF-A HIF response element (HRE) (Fig. 3C). Supershift EMSAs revealed Spry2 and HIF-1α but not HIF-2α or HIF-3α interacted at this complex (Fig. 3D). The VEGF-A promoter is regulated by HIF-1α and HIF-2α and so, to corroborate our EMSA results, we explored the effect of stable Spry2 shRNA knockdown in HBE at fetal PO_2_ on transcript expression of VEGF-A, BNIP-3 (HIF-1α-regulated) and CCND-1 (HIF-2α-regulated) (Preston et al., 2011). Spry2 knockdown significantly increased VEGF-A and BNIP3 transcript abundance with no overall effect on HIF-1α mRNA expression. CCND-1 expression was significantly suppressed in Spry2 knockdown cells suggesting that the induction of VEGF-A is likely mediated by HIF-1α (Fig. 3E).

### 3.3 Spry2 interacts with the VEGF-A promoter in FDLE

**Fig. 4:**
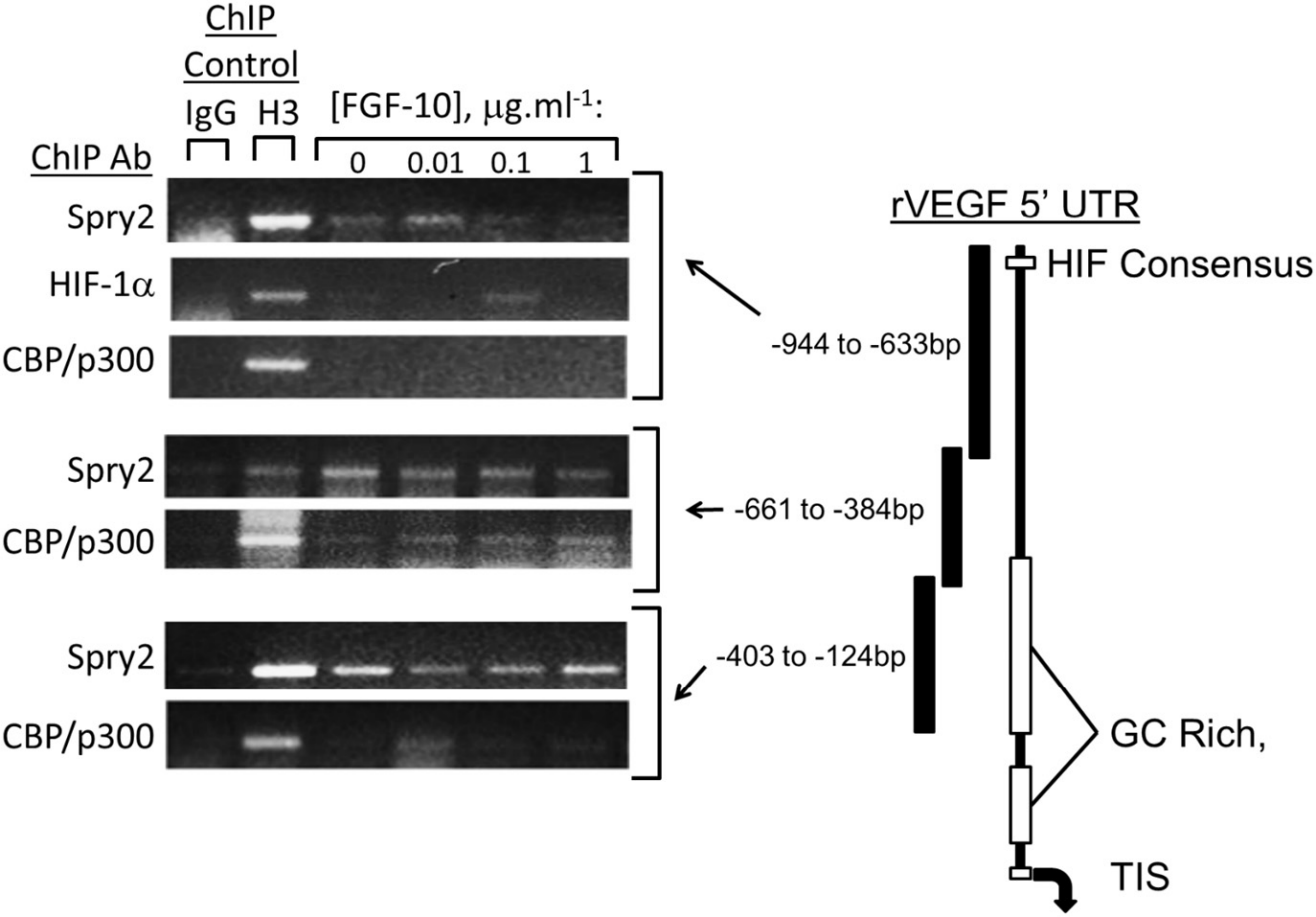
Nuclear Spry2 interacts with 5′ elements in the rat VEGF-A promoter. FDLE cells were incubated at fetal PO_2_ in the absence or presence of FGF-10 at the given concentrations. ChIP was performed using primers designed to amplify 5′ promoter elements as indicated in the diagram (right). Sequence alignments are to scale and are for the rat VEGF-A promoter; the position of the HIF consensus site is shown. Arrow at 3′ end indicates position of classical transcriptional initiation site (TIS). Result is representative of at least 4 independent experiments.

We hypothesised that nuclear Spry2 regulates the interaction between HIF-1α and the VEGF-A promoter during FGF-10 exposure and so chromatin immunoprecipitation (chIP) was used to determine if Spry2 interacts with VEGF promoter elements. The commercial Spry2 antibody (Abcam ab50317) was validated for this purpose (Supplementary Fig. 2) and used in combination with chIP grade antibodies against histone 3 (H3; positive control), HIF-1α and CBP/p300 (indicator of HAT binding and epigenetic gene activation). Parallel incubation with non-immune IgG served as a negative control. ChIP primers were designed against elements up to 1Kb upstream of the VEGF-A transcription initiation site (TIS) (Supplementary Table 2 and Fig. 4). In FDLE, nuclear Spry2 interacted with promoter elements spanning −944 to −633 bp (contains the rat VEGF-A HRE), −661 to −384 bp (contains the rat VEGF-A STAT-3 response element) and −403 to −124 bp (contains several GC rich domains) (Fig. 4). Primers designed against the transcription initiation site (TIS) failed to amplify in H3 positive control samples and so no conclusive observations were made at this location. Although chIP assays offer only qualitative assessment of protein-promoter interactions, we observed FGF-10 dependent interaction of nuclear Spry 2 and HIF-1α at the HRE-spanning region of the promoter and induced binding of CBP/p300 at the adjacent region spanning −661 to −384 bp. A strong, FGF-10-independent Spry2 signal was also observed at the most proximal region of the promoter investigated (−403 to −124 bp).

### 3.4 Conserved residues in the Spry2 cysteine rich domain (CRD) are necessary for interaction with the VEGF-A promoter

**Fig. 5:**
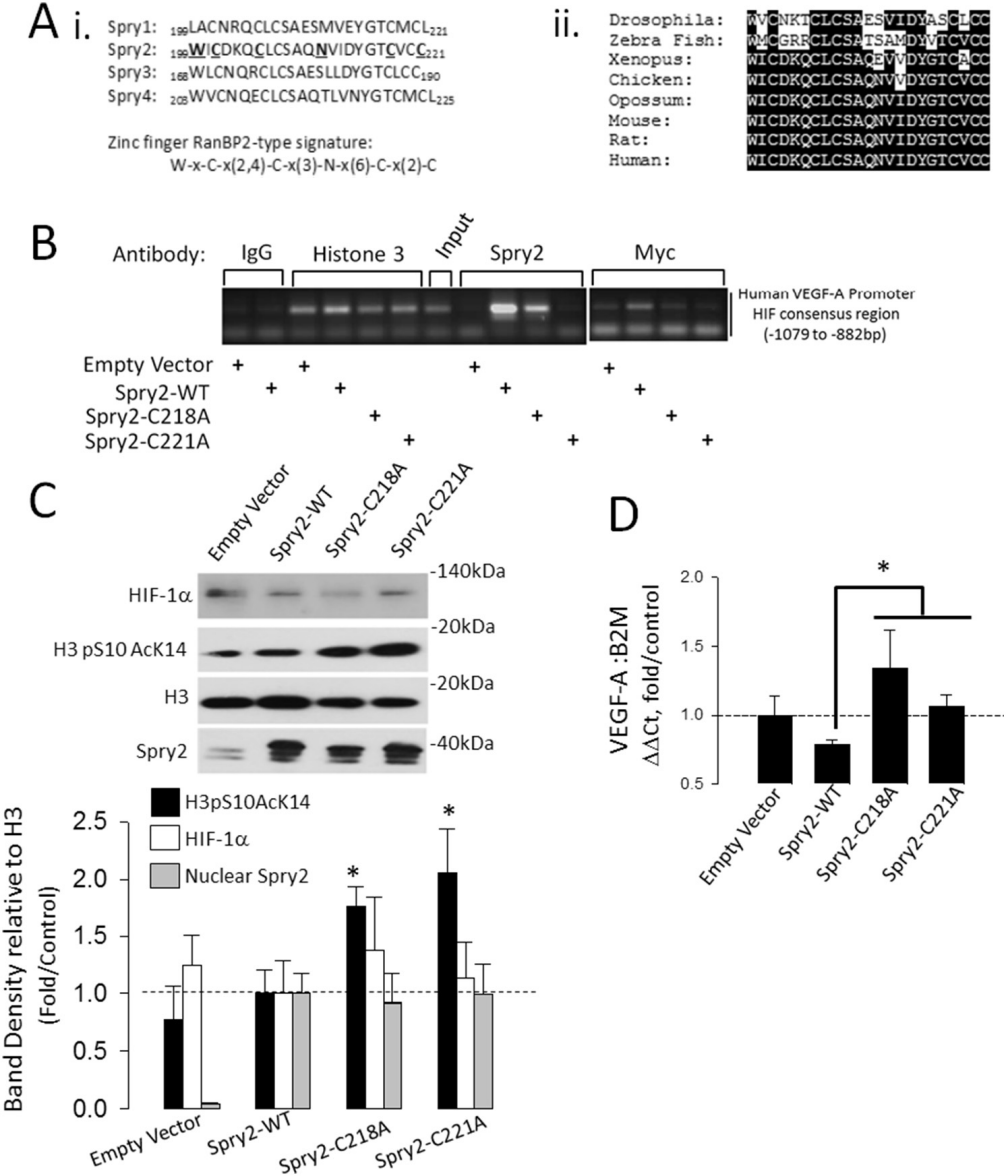
A region within the Spry2 cysteine rich domain (CRD) is necessary for Spry2 to interact with a region spanning the human VEGF-A HIF consensus site. A(i.) Sequence alignment of amino acids 199–221 in human Spry2 with Spry1, 3 and 4 with residues in Spry2 showing homology with the RanBP2 Zn^2+^ finger signature given in bold and underlined. (ii) Species conservation of the Spry2 sequence given in (i). B. ChIP assay against a region of the human VEGF-A promoter spanning the HIF consensus site. Stable HBE cell lines were generated that expressed either empty vector, wild-type Spry2 (Spry2-WT), Spry2-C218A or Spry2-C221A. ChIPs performed with Anti-Spry2 or Anti-Myc (C-terminal epitope) were performed in independent samples (2 each). C. HIF-1α and Spry2 nuclear abundance are unaffected but Histone-3 S10K14 phosphoacetylation is enhanced in stable HBE cells expressing C218A or C221A Spry2 mutants. *P < 0.05 relative to Spry2-WT, n = 5 D. qPCR showing augmented VEGF-A mRNA expression in HBE cells bearing Spry2-C218A or Spry2-C221A mutants. Values are mean ± SEM, n = 4, *P < 0.05 relative to Spry2-WT.

To determine how Spry2 might interact with DNA, bioinformatic analysis (ExPASy PROSITE) was performed to search for possible conserved DNA binding elements in the Spry2 protein sequence. This analysis identified limited homology between amino acids 199 and 221 in the Spry2 CRD and a zinc finger RanBP2-type signature (W-x-C-x(2,4)-C-x(3)-N-x(6)-C-x(2)-C; Entry Name: ZF_RANBP2_1, ExPASy Accession number PS01358). The corresponding sequence in Spry2 (_199_WICxxxCxCxxxNxxxxxCxxC_221_) is approximated in the same region between each of the other human Spry isoforms and is conserved between species (Fig. 5A). To determine if this region alters Spry2 interaction with the HIF consensus region of the human VEGF-A promoter, we mutated cysteines to alanines at position 218 or 221 (_199_WICxxxCxCxxxNxxxxxC/AxxC/A_221_), created stable HBE cell lines and performed ChIP assays using primers designed to amplify −1089 to −882 bp of the human VEGF-A promoter (Human VEGF-A HRE lies at −975 to −968 bp, (Pagès and Pouysségur, 2005)). ChIP was performed using anti-Spry2 or anti-myc antibodies to resolve the c-terminal epitope Myc tag of Dual tagged Spry2. IgG and H3 controls were performed as before. Both antibodies resolved interaction of wild-type (WT) Spry2 with this region of the VEGF-A promoter which was diminished or absent in cells stably expressing either C218A or C221A Spry2 mutants (Fig. 5B). Neither HIF-1α stability nor Spry2 nuclear abundance were altered by these treatments but C218A and C221A mutation significantly enhanced H3 S10K14 phosphoacetylation and raised VEGF-A expression compared to cells wild-type Spry2 transformed cells (Fig. 5C and D). These events occurred without detectable variation in the cellular distribution of wild-type, C218A or C221A Spry2 or endogenous ERK1/2 activity (Supplementary Fig. 3) suggesting that the mutant forms did not alter the non-nuclear function of Spry2. Thus, our data show that Spry2 is present in the nucleus of primary fetal, and immortalised adult, airway epithelial cells, interacts with discrete regions of DNA to influence gene expression (VEGF-A in this case) and that this interaction requires cysteine residues C218 and C221 in the Spry2 CRD.

### 3.5 Spry2 knockdown or CRD mutation diminishes CpG island methylation in the VEGF-A promoter

**Fig. 6:**
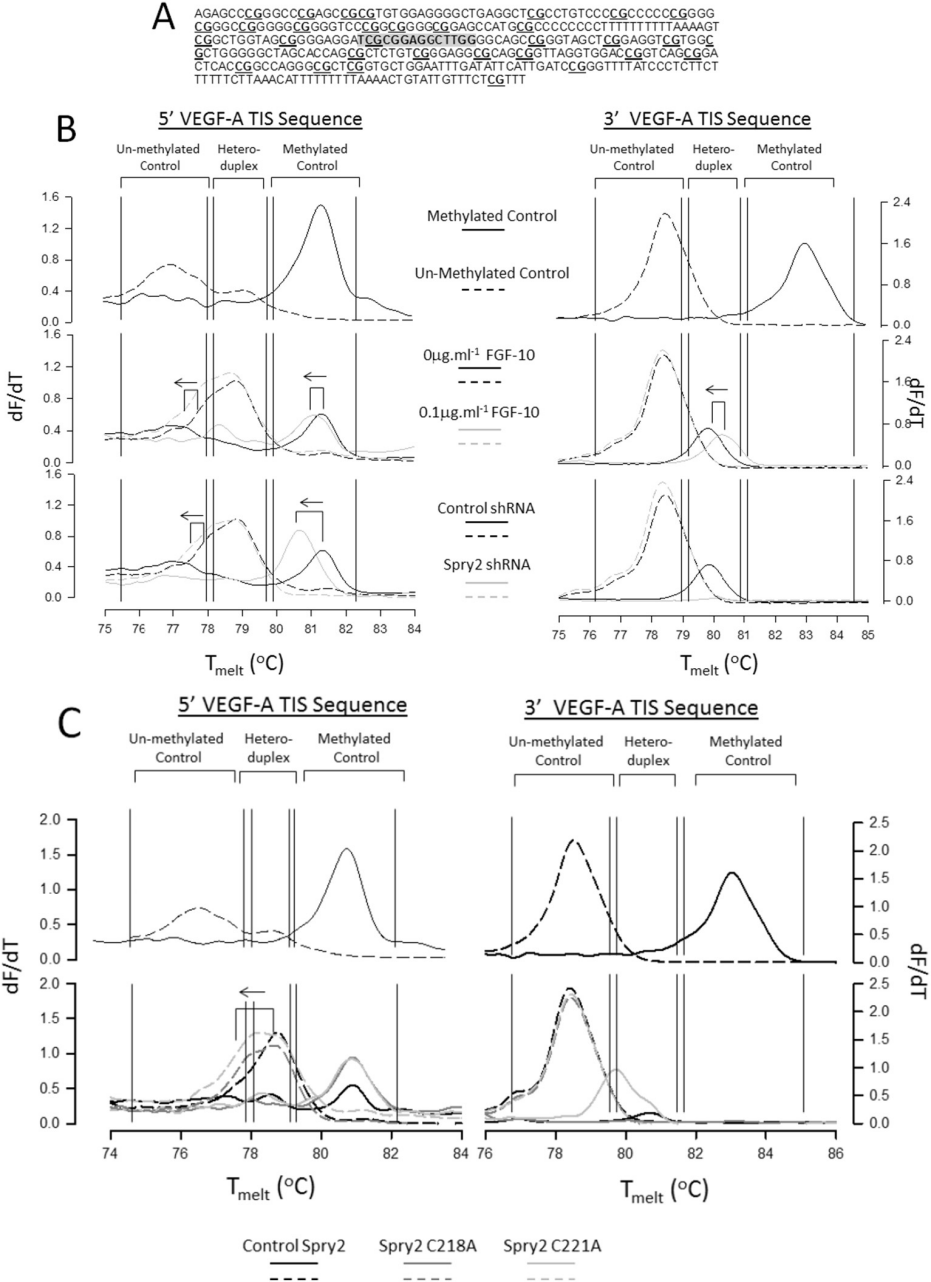
Manipulation of Spry2 function by FGF-10, shRNA knockdown or CRD mutation promotes de-methylation of the regions flanking the VEGF-A TIS in HBE cells. A. Sequence of the region flanking the human VEGF-A TIS (grey). Putative CpG methylation sites are highlighted (underlined) B. High resolution melt (HRM) analysis of VEGF-A promoter methylation. Control melt curves for 5′ and 3′ VEGF-A flanking sequences are shown at the top and were generated using 100% methylated (solid line) or 100% non-methylated DNA (dashed line). Corresponding melt curves for FGF-10 treated samples and control versus Spry2 shRNA are shown beneath respectively. Solid line: primers against methylated DNA; Dashed line: primers against unmethylated DNA. Leftward shift in HRM curve (arrows) indicates lowered incidence of CpG methylation. Peaks in heteroduplex region suggest non-uniform CpG methylation between VEGF-A alleles. C. As for panel A but relationship is shown for HBE cells stably expressing control (wild-type) Spry2, Spry2 C218A and Spry2 C221A. Graphs are representative of 3 independent experiments.

Spry2 knockdown and mutation of CRD residues C218 and C221 augment VEGF-A expression suggesting a role in silencing gene activity. CpG island methylation is an important part of this regulation and the region surrounding the transcriptional initiation site (TIS) of the human VEGF-A gene is rich in potential CpG methylation sites (Fig. 6A). We therefore used the Methylation Specific High Resolution Melt analysis (MS-HRM) method (Candiloro et al., 2011) to investigate the effect of FGF-10, Spry2 knockdown and Spry2 CRD mutation upon the methylation of CpG sites the VEGF-A TIS.

Two primer sets designed to detect methylated or unmethylated DNA sequences were used to analyse the methylation status of a region spanning −143 to 0 bp upstream of the VEGF-A site (5′VEGF-A TIS containing 17 CpG sites) and a second region spanning +13 to +199 bp downstream to the VEGF-A TIS (3′VEGF-A TIS containing 16 CpG sites). 20 ng of bisulfite converted DNA was amplified through 40 cycles of qPCR followed by HRM. Commercial methylated and unmethylated DNA served as controls.

5′VEGF-A TIS methylated primers resolved melt-peaks in the 0 μg·ml^−1^ FGF-10-treated and shRNA control samples that corresponded to the position of the 100% methylated control indicating extensive methylation of this region of the VEGF-A promoter. Treatment with FGF-10 or knockdown of Spry2 induced a leftward shift toward the position of the unmethylated control peak (Fig. 6B). Primers designed to detect unmethylated DNA within this region revealed that most of the DNA in this region occurs as a heteroduplex suggesting differential methylation occurs between complementary DNA strands, however, both FGF-10 treatment and Spry2 knockdown raised the proportion of the DNA melt curve that fell under the unmethylated control peak. In contrast, the vast majority of DNA amplified from the 3′ TIS region resolved as un-methylated DNA and was not affected by any of the treatments. Taken together, these results indicate that FGF-10 treatment or Spry2 shRNA knockdown results in de-methylation of CpG residues in the 5′VEGF-A TIS. A similar analysis of the 3′VEGF-A TIS indicated that, although heterogeneous DNA methylation was evident with the methylated primer sets, the majority of CpG sites in this region remained unmethylated.

Mutation of C218 and C221 residues in the CRD region of Spry2 also altered DNA methylation patterns in the 5′VEGF-A TIS region (Fig. 6C). Although no shift in the methylated peak position was observed, C218A or C221A mutation increased the proportion of the unmethylated primer product under the peak area of the unmethylated control. Once again, very little DNA methylation was detected in the 3′TIS VEGF-A region. Taken together with the loss of DNA interaction and increased VEGF-A expression, the mutation of these residues appears critical for Spry2 DNA interactions and epigenetic regulation of gene expression.

## 4 Discussion

The semi-fractal organisation of the pulmonary tree, as beautifully illustrated by Weibel's latex cast, implies the presence of a molecular branching periodicity regulator which, 1) sets the duration of tubular elongation, 2) determines the position of each branch point, 3) re-calibrates branching subroutines at each daughter generation, 4) co-ordinates all of these processes between airway and vascular systems and 5) determines the point at which the branching programme diverges to form the gas exchange surfaces. Here, we report that Spry2, a conserved regulator of branching morphogenesis across species, is present in the nucleus of airway cells derived from the fetal lung, is regulated by the primary inducer of airway outgrowth, FGF-10, and represses the activity of the main inducer of VEGF-A transcript expression, HIF-1α. Using chIP and DNA methylation analysis, we show that Spry2 interacts directly with regulatory regions of the VEGF-A promoter and that its knockdown induces demethylation and subsequent expression of VEGF-A transcripts and protein. Taken as a whole, we argue that Spry2 has a central role to play in the molecular co-ordination of airway and vascular branching periodicity. As such, it is an important contributor to design optimality in the cardio-pulmonary system.

The mechanisms which underpin nuclear Spry2 signalling require evaluation. The electrophoretic migration of Spry2 as a doublet is frequently reported in the literature (see, for examples (Anderson et al., 2011; Aranda et al., 2008; Fong et al., 2003; Impagnatiello et al., 2001; Scott et al., 2010)) and a number of processes could account for this difference in molecular weight. Spry2 is post-translationally modified by phosphorylation and palmitoylation (Impagnatiello et al., 2001) and is also ubiquitylated through its interaction with E3 ligases such as Cbl, SIAH2 and NEDD4 (reviewed Edwin et al., 2009). Although our results show evidence that Spry2 is palmitoylated and that its protein abundance is stabilised by kinase inhibition, neither effect accounted for the molecular mass difference between the two forms. One possibility, untested here, is that the 39 kDa form represents a mono-ubiquitylated form of Spry2 that is primed to respond to growth factor signals. Lysine monoubiquitylation has been linked to protein trafficking between compartments and organelles and is distinct from polyubiquitylation in that it does not necessarily result in protein degradation. In some circumstances, this process promotes transcription factor nuclear translocation and DNA binding (e.g. FOXO4 (van der Horst et al., 2006)) and within the nucleus itself, histone monoubiquitylation governs the epigenetic status of DNA (Ouni et al., 2011). Given that Spry2 interacts with ubiquitin ligases and is known to translocate between different compartments of the cell, it is possible that the molecular weight differences are driven primarily by monoubiquitylation and secondarily by polyubiquitylation that controls the overall abundance of the Spry2 protein.

Repression of vascular signalling by Spry proteins has been described in a number of models of development and disease and underpins the role of these proteins as critical mediators of vascular stability. For example, Spry2 or 4 knockout in mice causes malformation of cardiovascular structures that is lethal at birth and shRNA knockdown of these proteins in a murine model of hind-limb ischemia-reperfusion injury drives angiogenesis following ischemic injury (Taniguchi et al., 2009). In lung development and wound repair, high Spry2 expression levels correlate with vascular atrophy (Lazarus et al., 2011; Wietecha et al., 2011). These effects are explained, at least partly, by Spry2-mediated repression of angiogenic RTK signalling along the ras/MEK/ERK1/2 pathway, however, to our knowledge, no study has investigated the possibility that Spry2 may directly influence the process of vascular gene activation in the nucleus. Our results support a role for Spry2 as a repressor of transcriptional events that control vascular gene expression and that RTK signalling (in this case, activation of FGFR2b by FGF-10) relieves this by clearing the 39 kDa form of Spry2 from the nucleus.

HIF-1α mediates vascular gene expression in the early stages of lung development and so we tested the hypothesis that repression of HIF activity and VEGF-A expression was mediated by interaction between Spry2 and the VEGF-A promoter to alter one of, i) HIF-1α recruitment to the HRE, ii) CBP/p300 histone acetyl transferase recruitment or iii) DNA methylation status. We found that Spry2 interacted to varying extents with regions of the rat VEGF-A promoter spanning −944 bp to −142 bp. Reliable resolution of protein-DNA interactions could not be detected at sites flanking the transcription initiation site (TIS) because the high GC content this region. Notably, however, we observed a FGF-10-responsive interaction in the region of the promoter that spanned the HRE and apparently constitutive interactions in neighbouring downstream regions that include response elements for STAT3 and SP1/SP3. In FDLE cells, the loss of Spry2-DNA interaction that occurred with FGF-10 treatment corresponded with a transient increase in the HIF-1α-binding at this site and also increased VEGF-A expression. This differed in HBE cells, however, where stable knockdown of Spry2 produced similar increases in VEGF-A expression but did not alter HIF-1α or CBP/P300 binding to this region. In FDLE, the CBP/p300 interaction was detected in the neighbouring downstream site and increased with the decline in Spry2 DNA binding. Since STAT3 interacts with this site and is known to modulate HIF-1α signalling and VEGF-A expression (Xu et al., 2005) we investigated if the activity of this transcription factor was altered by changes Spry2 abundance. We found no significant activation of STAT3 with any of these treatments (data not shown) and so excluded this pathway from further investigation. In HBE cells, our investigation of the human VEGF-A HRE region revealed a number of important functional aspects of the Spry2 interaction at this region. Firstly, we found that Spry2 knockdown abolished the oxygen dependence of HIF-1α signalling and profoundly raised both HIF-1α and CBP/p300 interaction with this site. This was accompanied by increased HIF-1α activity in high oxygen conditions. Secondly, we showed that the interaction of Spry2 at this site depended upon conserved cysteine residues at position 218 and 221 since mutation of either residue resulted in loss of Spry2 interaction and increased HIF-1α activity and VEGF-A expression. These residues were identified from a PROSITE scan of homologous Zn^2+^ finger domains and although we did not investigate if Zn^2+^ was necessary for the Spry2-DNA interaction, it is possible that this site acts as a redox centre that mediates Spry2 interaction with other macromolecules, including DNA. Indeed the high level of conservation of these residues from insects to mammals and between mammalian Spry isoforms suggests that they are critical mediators of Spry2 function. Taken together, however, these results suggest that Spry2 functions as a repressor of VEGF-A expression, possibly by controlling the recruitment of HIF-1α and CBP/p300 transcription co-activators to the VEGF-A promoter.

The 5′ TIS region of the VEGF-A promoter is flanked by GC rich sequences whose methylation may regulate transcription of the VEGF-A gene. We were unable to reliably detect Spry2 interactions in this region using ChIP and so high resolution melt analysis (Candiloro et al., 2011; Xu et al., 2005) was used to determine if CpG methylation was altered by FGF-10, Spry2 shRNA or Spry2 C218/C221 mutation. H3-S10/K14 phosphoacetylation blocks methylation of H3-K9, a marker of gene condensation and lowered gene expression (Candiloro et al., 2011), and was dose-dependently increased by FGF-10. In keeping with this, we detected methylated and heteroduplex (methylated/unmethylated alleles) in all samples and found that FGF-10 and Spry2 knockdown resulted in a leftward shift in melting profile that is consistent with a de-methylation of the VEGF-A promoter in this region. Methylated DNA was not detected in the downstream 3′TIS region. Similarly, stable mutation of cysteines 218 and 221 produced a leftward shift in heteroduplex methylation compared to wild-type Spry2. As a whole, this data suggests that the presence of Spry2 in the nucleus is associated with VEGF-A promoter methylation and events which disrupt its function (FGF-10, shRNA C218, C221 mutation) result in loss of methylation and induction of the gene.

**Fig. 7:**
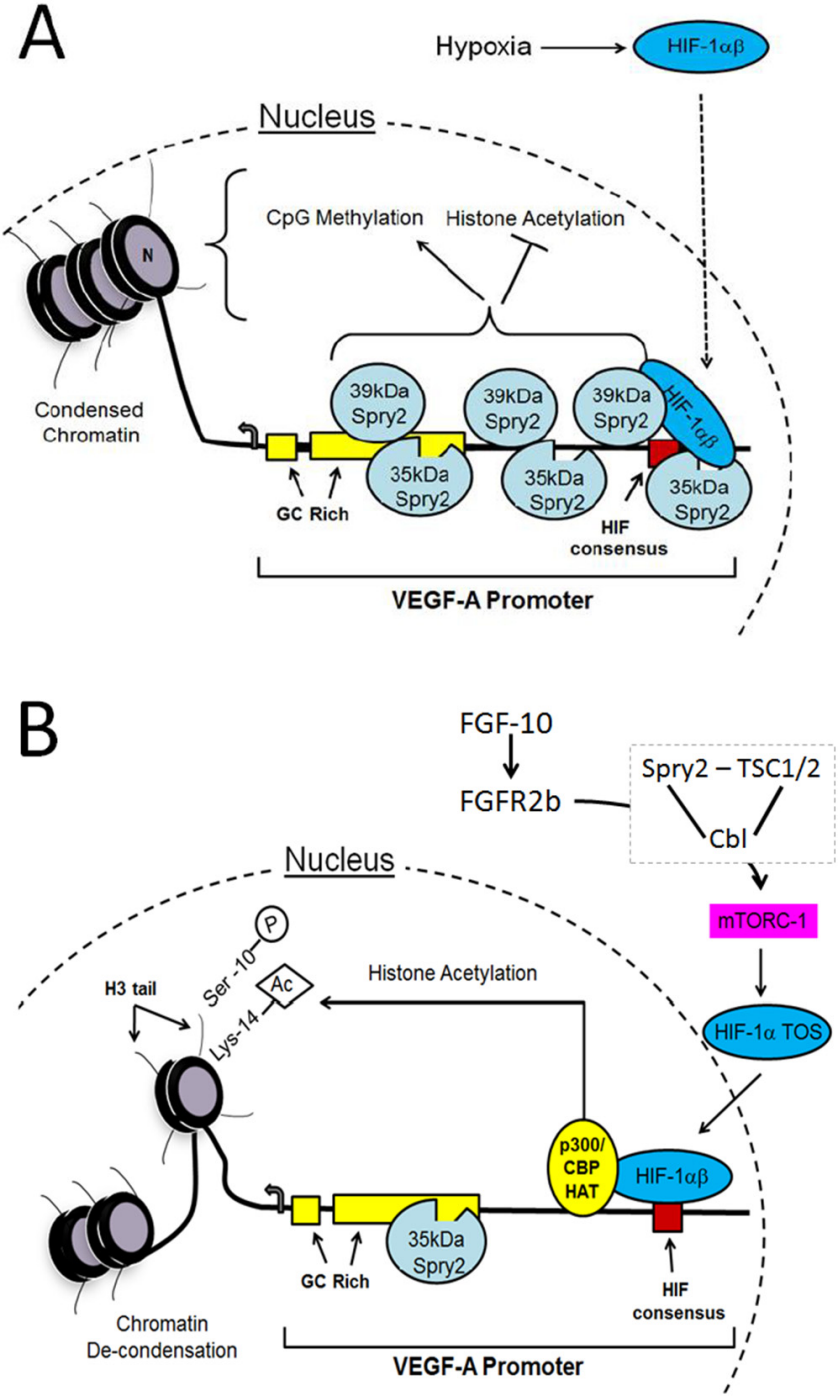
Proposed model of Spry2-regulated VEGF-A gene expression. A. Hypoxic induction of HIF-1α is held in check by interaction of high and low molecular weight Spry2 isoforms with 5′ regions of the VEGF-A promoter. Under these conditions HIF transcriptional activation is rendered inefficient due to weak binding to CBP/P300 (Impagnatiello et al., 2001; Preston et al., 2011), CpG island methylation, histone deacetylation and DNA condensation (this study). B. FGF-10 signalling through its receptor, FGFR2b, induces Spry2- and Cbl-dependent clearance of the mTOR repressor complex, TSC1/2. The active mTOR complex 1 (mTORC1) binds to an mTOR binding motif (TOS) on HIF-1α and increases the efficiency of HIF-driven gene expression by promoting its interaction with p300/CBP (Impagnatiello et al., 2001; Land et al., 2014; Land and Tee, 2007; Scott et al., 2010). VEGF-A promoter activity is raised by clearance of Spry2 from non GC-rich regions including the HRE; Spry2 that remains bound to GC rich regions will be in the 35 kDa form. Subsequent histone phospho-acetylation and chromatin de-condensation augments VEGF-A gene expression in proportion to growth factor signal strength.

Fig. 7 places these data into context with the mechanisms which link Spry2 to the control of vascular gene signalling in the fetal lung. In the absence of FGF-10, hypoxic induction of the VEGF-A gene is held in check by interaction of the 39 and 35 kDa forms of Spry2 with regions of the 5′VEGF-A promoter over several transcriptional regulation sites including the HRE. FGF-10 relieves this interaction in two ways. Firstly, our previous results demonstrate that loss of 39 kDa Spry2 in the cytosol promotes HIF-1α activation by favouring mTORC-1-dependent interaction between HIF-1α and its transcriptional co-activator, CBP/P300 (Land et al., 2014; Scott et al., 2010). Secondly, the present data adds to this model by showing that loss of 39 kDa Spry2 from the nucleus promotes de-condensation of DNA and so facilitates growth factor dependent VEGF-A gene expression. These results add to the established role of Spry2 as a modulator of receptor tyrosine kinase signalling, and important contributor in disease (Edwin et al., 2009), by demonstrating that this protein co-ordinates nuclear gene activation events with prevailing growth factor conditions. In comparative terms, the scene is set to expand investigation of sprouty proteins and related forms (e.g. SPREDs) to understand the evolutionary origins of branching morphogenesis programmes, from the excretory canal cell of nematodes to complex structures including the human brain.

## Appendix A Supplementary data

Supplementary material Image 1
