# Supplementary material for: Regulation of vascular signalling by nuclear Sprouty2 in fetal lung epithelial cells: Implications for co-ordinated airway and vascular branching in lung development
Source: Comp Biochem Physiol B Biochem Mol Biol. 2018 Oct;224:105–14. doi: 10.1016/j.cbpb.2018.01.007 (PMC6078907; doi:10.1016/j.cbpb.2018.01.007)
Supplement: Supplementary file 1 — Supplementary material [file mmc1.docx]

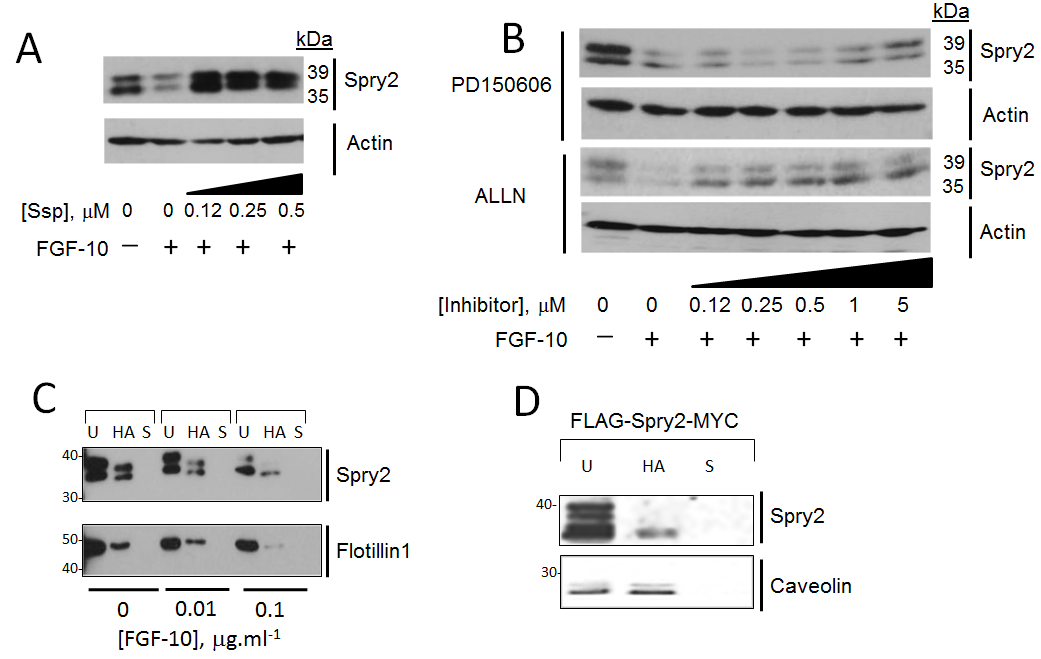
**Supplementary Figure 1:**

**Supplementary Figure 1. Phosphorylation, calpain activity and palmitoylation alter Spry2 abundance and function but do not account for the difference in mass between 35 and 39kDa Spry2. A.** FDLE cells treated with staurosporine (Ssp) at concentrations shown for 2h in presence or absence of FGF-10. Ssp treatment increases the abundance of both Spry2 forms. Representative of 4 independent experiments. **B.** FDLE cells treated with calpain inhibitors, PD150606 or ALLN at concentrations shown for 2h in presence or absence of FGF-10. Both inhibitors increase the abundance of both Spry2 forms. Representative of 4 independent experiments. **C.** Protein S-Acylation by resin assisted capture (Acyl-RAC) resolves high and low molecular weight forms of palmitoylated Spry2 in FDLE which decline with total Spry2 abundance on treatment with FGF-10. See below for method; U, unfractionated input, HA, hydroxylamine, S, saline negative control. Flotillin-1 palmitoylation serves as a positive control. Representative of 3 independent experiments. **D.** Acyl-RAC reveals that only the lower band of Dual-tagged Spry2 is palmitoylated in 16HBE14o- cells. Caveolin was used as a positive control. Representative of 3 independent experiments.

**Results:** Difference in Spry2 molecular weight in response to growth factor stimulation is a major correlate of Spry2 function and has been linked to phosphorylation, palmitoylation and proteolytic cleavage (1). We attempted to resolve which of these processes might account for the difference in molecular weight between the 35 and 39kDa bands in FDLE. Interaction between Spry2 and the cCBl ubiquitin ligase requires phosphorylation of the tyrosine kinase phosphorylation domain (Y55-TKD) and so inhibition of this effect might be expected to stabilise a lower molecular weight form of Spry2. The broad-spectrum kinase inhibitor, staurosporine, stabilised both forms of Spry2 in response to FGF-10 confirming our previous observation that this growth factor promotes its phosphorylation-dependent proteolytic clearance (**Supplementary Figure 1A**). The lack of selection of one form over the other suggests, however, that the molecular weight difference is not due to phosphorylation or associated proteolytic cleavage. Calpains can end-modify nuclear proteins to produce a molecular weight difference (2) and so we used PD150606 (Ki Calpain I = 201nM; Calpain II = 370nM) and N-Acetyl-L-leucyl-L-leucyl-L-norleucinal (ALLN; Ki Calpain I = 190nM; Calpain II = 220nM) to determine if FGF-10 induces cleavage of Spry2 by this mechanism. As with staurosporine, both calpain inhibitors dose-dependently recovered Spry2 abundance in response to FGF-10 but did not significantly alter the ratio of high:low molecular weight Spry2 isoforms (**Supplementary Figure 1B**). Moreover, our finding that FLAG and MYC antibodies both resolved N and C-terminally tagged Spry2 at the same molecular weight in the nucleus also confirms that end-cleavage of Spry2 does not account for the disappearance of high molecular weight bands (**Figure 1C**). Palmitoylation of Spry2 was investigated using resin-assisted capture of S-acylated cysteine residues (Acyl-Rac) because the addition of lipid moieties to nuclear proteins can alter protein-DNA interactions (3,4). Palmitoylated Spry2 was resolved in FDLE cells at 35 and 39kDa in FDLE and FGF-10 induced the clearance of the 39kDa form independently of its palmitoylation status. In HBE, palmitoylated Spry2 resolved exclusively at 35kDa suggesting that this post-translational modification does not account for the higher molecular weight bands of this protein (**Supplementary Figures 1C and D**). Overall, the molecular weight differences in Spry2 observe in FDLE and HBE could not be attributed post-translational modification by phosphorylation, palmitoylation or proteolytic cleavage but we note that responses to these events are conserved between the two Spry2 forms.

**Method:** Protein S-Acylation by resin assisted capture (Acyl RAC) was performed according to Forrester et al, 2011 (4). Briefly, protein was acetone extracted from 2x10^6^ cells following blocking of free thiol groups with methyl methanethiosulfonate (MMTS). The protein extract was washed and re-suspended in a binding buffer containing (in mM): 100 HEPES (pH7.5), 1 EDTA and 1% w/v SDS. A proportion of the lysate was saved at this point as the total input or unfractionated (U) sample. The remaining lysate was incubated on a rotator for 2.5h with thiopropyl sepharose beads (Sigma T8387) at a volume ratio of 1:1 in the presence of either 1M (final conc) NH_2_OH (hydroxylamine, HA) or 1M (final conc) NaCl (S) at pH 7.5. The beads were then washed 5 times in 1ml binding buffer and incorporated proteins were eluted at 60^o^C using Laemmi sample buffer supplemented with 100mM ditiothreitol. Samples were run on SDS-PAGE gels and western blotted for Spry2. Flotillin-1 or Caveolin were used as positive controls for palmitoylation.

References:

1. **Edwin F, Anderson K, Ying C, Patel TB**. Intermolecular interactions of Sprouty proteins and their implications in development and disease. Mol Pharmacol. 2009; 76(4):679-91.
2. **Tremper-Wells B, Vallano ML.** Nuclear calpain regulates Ca2+-dependent signaling via proteolysis of nuclear Ca2+/calmodulin-dependent protein kinase type IV in cultured neurons. J Biol Chem. 280(3):2165-75.. Oncogene. 2005; 24(36):5552-60.
3. **Forrester MT, Hess DT, Thompson JW, Hultman R, Moseley MA, Stamler JS, Casey PJ.** Site-specific analysis of protein S-acylation by resin-assisted capture. J Lipid Res. 2011; 52(2):393-8.
4. **Wilson JP, Raghavan AS, Yang YY, Charron G, Hang HC.** Proteomic analysis of fatty-acylated proteins in mammalian cells with chemical reporters reveals S-acylation of histone H3 variants. Mol Cell Proteomics. 2010; 10(3):M110.001198

**
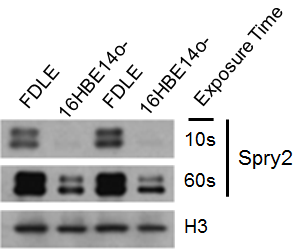
Supplementary Figure 2.**

A

B


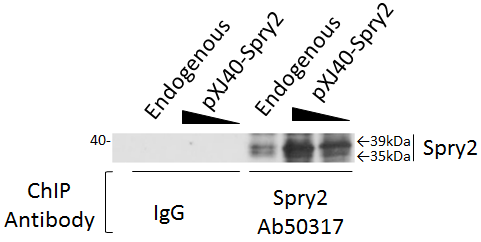


**Supplementary Figure 2. A.** Native nuclear Spry2 expression in FDLE and 16HBE14o- (HBE) cells. Two independent experiments are shown with the same blots exposed to film for either 10 or 60 secs. H3, histone 3. **B.** Recovery of endogenous and over-expressed Spry2 from formaldehyde cross-linked HBE cells using rabbit Anti-Spry2 (Abcam 50317). 1μg of antibody was used to immunoprecipitate Spry2 from cells that had been cross-linked for ChIP analysis. An equivalent amount of non-immune anti-rabbit IgG was used as a negative control Cells were either untransfected (endogenous) or transfected with two concentrations of pXJ40-Spry2 and then processed for ChIP to the cross-link reversal step. Protein recovered at this stage was western blotted for Spry2.

**Method 1: Preparation of Cytosolic, Nuclear and Chromatin-bound lysates.:** All initial washing and lysis stages were conducted in a hypoxic chamber maintained at the PO_2_ of the fetal lung (3% O_2_). Control and FGF-10 treated cells were washed twice in ice cold, O_2_- equilibrated phosphate buffered saline (PBS) and then scraped into same, containing 10µM MG-132 before being gently sheared by 20 passes through a 100μl pipette tip. The resulting slurry was centrifuged at 13,000xg for 5 minutes and the remaining pellets were re-suspended in 200μls of Cytosolic Lysis Buffer (25mM HEPES, 5mM KCl, 0.5mM MgCl2, one Complete protease inhibitor tablet and 10µM MG-132), sheared as before and then vortexed on an IKA Vibrax for 15 minutes with 1µl of NP-40 added to each suspension at the 10th minute of shaking. The vortexed suspensions were then centrifuged at 13000xg for 3 minutes and supernatants were retained as the cytosolic fractions. The pellets were re-suspended in 100μls of Nuclear Lysis Buffer (25mM HEPES, 10% (w/v) Sucrose, 350mM NaCl, 0.01% (w/v) NP-40, one Complete (Roche) protease inhibitor cocktail tablet and 10µM MG-132) and sheared for a third time as above. Complete disruption was achieved by sonicating each sample on an ice-slush slurry using a Soniprep 150 sonicator set at an amplitude of 6μm for two, 10s bursts. Lysates were centrifuged at 13,000xg for 10 mins and supernatants were retained as the nuclear fraction. Protein content of cytosolic and nuclear fractions was determined using the Bradford assay. The remaining pellets were taken as the insoluble chromatin fraction and were lysed directly in 50μls of Laemmi sample buffer.

**Method 2: Chromatin Immunoprecipitation:** FDLE or HBE cells were washed twice in ice-cold PBS following experimental treatments and cross-linked with 1% formaldehyde for 10 minutes. The reaction was quenched with 2.5M glycine and cells were then washed twice and scraped into PBS into a sterile Eppendorf. The pellet was re-suspended in SDS lysis buffer (50mM Tris-HCl pH 8.1, 10mM EDTA, 1% SDS and one Complete (Roche) protease inhibitor cocktail tablet) and left on ice for 10 minutes. Lysates were sheared ten times at an amplitude of 10 microns for 15 seconds per burst using a Soniprep 150 sonicator. The supernatant was recovered and diluted 1:10 with ChIP dilution buffer (16.7mM Tris-HCl pH 8.1, 167mM NaCl, 1.2mM EDTA, 0.01% SDS, 1.1% Triton X-100). 20µl was removed for use as an ‘input control DNA’. The remainder of the lysates were pre-cleared with salmon sperm DNA/Protein A Agarose-50% slurry (Millipore; 16-157C) for 30 minutes at 4°C and 2µg of immunoprecipitating antibody was added to the recovered supernatant and incubated overnight at 4°C. For a negative and positive control, 1μg of non-immune murine IgG and H3 antibody were used respectively. Salmon Sperm DNA/Protein A Agarose was added to pull down the antibody/histone complex for 1 hour and the pellets were washed once with the following buffers; Low Salt Immune Complex Wash Buffer (20mM Tris-HCl pH 8.1, 150mM NaCl, 2mM EDTA, 0.1% SDS , 1% Triton X-100), High Salt Immune Complex Wash Buffer (20mM Tris-HCl pH 8.1, 500mM NaCl, 2mM EDTA, 0.1% SDS, 1% Triton X-100), LiCl Immune Complex Wash Buffer (10mM Tris-HCl pH 8.1, 1mM EDTA, 0.25M LiCl, 1% IGEPAL-CA630 , 1% deoxycholic acid) and two washes in TE buffer (10mM Tris-HCl and 1mM EDTA, pH 8.0). Lysates were then twice eluted from the beads with ChIP elution buffer (0.1M NaHCO_3_, 1% SDS) and the crosslinks were reversed overnight at 65°C with addition of 5M NaCl. Lysates were incubated at 55°C for 1 hour with addition of 0.5M EDTA, Tris-HCl pH 6.5 and 10mg/ml Proteinase K and heat-shocked for 10 minutes at 100°C. DNA was extracted using a Nucleospin Gel and PCR clean-up kit (Macherey-Nagel) according to the manufacturer’s instructions. The immunoprecipitated DNA was analysed by qPCR under reaction conditions of 95°C for 7s and 60°C for 14s. DNA was run on a 1% agarose gel and visualised by UV. The primers used to amplify regions of the r/hVEGFA promoter encompassing the HIF response element (HRE), STAT response element (SRE) and distal GC rich response elements are listed in **Supplementary Table 2**. The identity of amplified products was confirmed by sequencing.

Supplementary Figure 3.


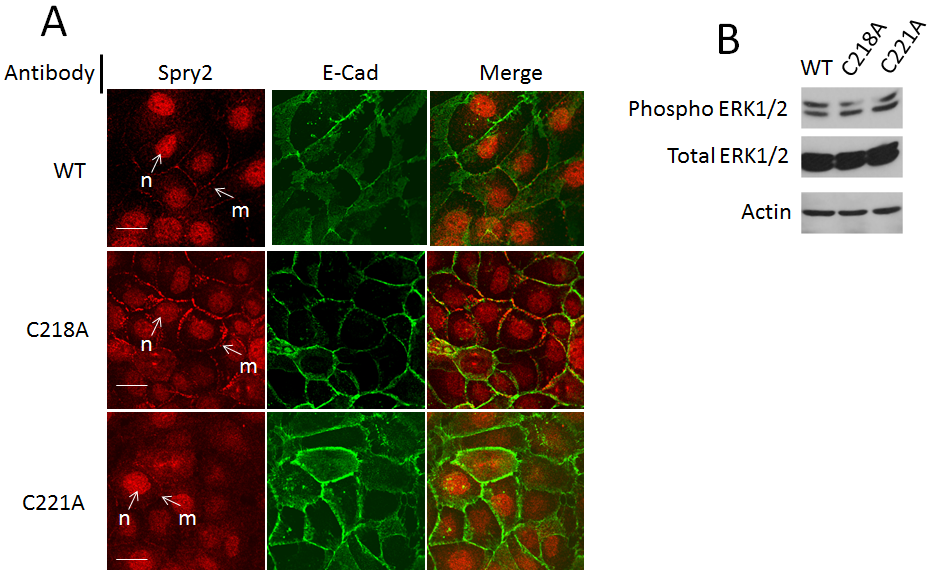


**Supplementary Figure 3. A.** Intracellular distribution of Spry2 is not significantly altered in HBE cells stably expressing wild-type (WT), C218A and C221A Spry2. Method: Cells were grown in 8-well chambers and processed for immunofluorescence using antibodies against Spry2 (1:1000, Abcam) or E-Cadherin (1:1000, Abcam). Images are representative of two independent experiments. **B.** Endogenous ERK1/2 activation is not altered in HBE cells stably expressing the same Spry2 variants. Blot is representative of two independent experiments.

**Supplementary Table 1.**

**Table 1.1 primary antibodies used in this study.**

| **Primary Antibody** | **Species** | **Supplier** | **Dilution Factor/Concentration** | **Molecular Weight (kDa)** |
| --- | --- | --- | --- | --- |
| Acetylated α Tubulin | Mouse | Abcam  (ab24610) | 1:2000 (IF) | 55 |
| ß-Actin | Rabbit | NEB (4970) | 1:5000 (WB) | 45 |
| Caveolin | Rabbit | Abcam  (ab2910) | 1:1000(WB); 1μg (IP) | 22 |
| CBP/p300 | Mouse | Abcam (ab14984) | 1:1000 (WB)/2µg (ChIP) | 300 |
| DYKDDDDK-Tag  (FLAG epitope) | Rabbit | Cell Signaling | 1:1000 (WB); 1:500 (IF) | N/A |
| E-Cadherin | Mouse | Abcam  (ab1416) | 1:1000 IF | N/A |
| Flotillin-1 | Rabbit | Abcam  (ab41927) | 1:1000(WB); 1μg (IP) | 47 |
| HDAC1 | Mouse | Abcam (ab46985) | 1:1000 (WB) | ~55-57 |
| HDAC2 | Rabbit | Abcam (ab32117) | 1:1000 (WB) | ~55-60 |
| HDAC3 | Rabbit | Abcam  (ab16047) | 1:1000 (WB) | 49 |
| HIF-1α | Rabbit | Abcam | 1:1000 (WB)/2µg (ChIP) | 120 |
| HIF-2α | Goat | R & D Systems (AF2886) | 1:1000 (WB) | 115 |
| HIF-3α | Rabbit | Abcam (ab2165) | 1:1000 (WB) | ~72-80 |
| Histone H3 Total | Rabbit | NEB (2650) | 1:5000 (WB)/1µg (ChIP) | 17 |
| Histone 3 phospho (Ser10)-acetyl (Lys14)- | Rabbit | Millipore (07-081) | 1:1000 (WB) | 17 |
| Myc-epitope | Mouse | Life Technologies (46-0603) | 1µg (ChIP); 1:500 IF | N/A |
| P84 Nuclear Matrix | Mouse | Abcam (ab487) | 1:2000 (WB) | 84 |
| Sprouty 2 | Rabbit | Abcam (ab50317) | 1:1000/2000 (IF/WB), 2µg (ChIP), 1µg (Co-IP) | Doublet at 39 and 35 |

**Abbreviations: WB (Western blot), IF (Immunofluorescence), ChIP (Chromatin Immunoprecipitation), IP (Immunoprecipitation), NEB (New England Biolabs).**

**Table 1.2 Secondary antibodies used in this study.**

| **Secondary Antibody** | **Supplier** | **Dilution Factor/Concentration** |
| --- | --- | --- |
| Alexa Fluor 488 Donkey Anti-Mouse | Life Technologies (A21202) | 1:2000 (IF) |
| Alexa Fluor 555 Donkey Anti-Rabbit | Life Technologies (A31572) | 1:2000 (IF) |
| Alexa Fluor 680  Goat Anti Mouse | Life Technologies  (A21057) | 1:2000 (Licor) |
| IRDye800 conjugated AP Goat anti-rabbit | Rockland  611-132 | 1:2000 (Licor) |
| Anti-Biotin, HRP-linked | NEB (7075S) | 1:1000 (WB) |
| Anti-Mouse IgG, HRP-linked | NEB (7076S) | 1:1000 (WB) |
| Anti-Rabbit IgG, HRP-linked | NEB (7074S) | 1:1000 (WB) |

**Abbreviations: WB (Western blot), IF (Immunofluorescence), NEB (New England Biolabs).**

**Supplementary Table 2.**

Rat VEGF-A promoter ChIP primers.

| Rat VEGF-A 5’UTR | Forward Sequence (5’-3’) | Reverse Sequence (5’-3’) |
| --- | --- | --- |
| -994 to -633bp  (HRE region) | GATCAGGAGGAACAAGGGCTTCTG | GCAGGCTTTGACTTCCCAAATAG |
| -661 to -384bp  (STAT3 region) | GTTTCCGAGGTCAAACAAGC | CACACTATACCCAGACACAC |
| -403 to -124bp  (Distal region) | GTGTGTCTGGGTATAGTGTG | GCCACTACTGCGAAATAGAAA |

Human VEGF-A promoter ChIP primers.

| Human VEGF-A 5’UTR | Forward Sequence (5’-3’) | Reverse Sequence (5’-3’) |
| --- | --- | --- |
| -1080 to -833bp  (HRE region) | CCTCAGTTCCCTGGCAACATCTG | GGCACCAAGTTTGTGGAGCTGAG |
| -891 to -503bp  (STAT3 region) | TTGGTGCCAAATTCTTCTCCCCT | CACACGTCCTCACTCTCGAAGAC |
| -561bp to -205bp  (Distal region) | CACCACAGGGAAGCTGGGTGA | CCAAGGTTCACAGCCTGAAAATTAC |
